# Food, Dietary Patterns, or Is Eating Behavior to Blame? Analyzing the Nutritional Aspects of Functional Dyspepsia

**DOI:** 10.3390/nu15061544

**Published:** 2023-03-22

**Authors:** Charalampia Amerikanou, Stamatia-Angeliki Kleftaki, Evdokia Valsamidou, Eirini Chroni, Theodora Biagki, Demetra Sigala, Konstantinos Koutoulogenis, Panagiotis Anapliotis, Aristea Gioxari, Andriana C. Kaliora

**Affiliations:** 1 Department of Nutrition and Dietetics, School of Health Science and Education, Harokopio University, 70 El. Venizelou Ave, 17676 Athens, Greece; amerikanou@windowslive.com (C.A.); matina.kleftaki@gmail.com (S.-A.K.);; 2 Department of Nutritional Science and Dietetics, School of Health Science, University of the Peloponnese, Antikalamos, 24100 Kalamata-Messinia, Greece

**Keywords:** functional dyspepsia, epigastric pain syndrome, postprandial distress syndrome, foods, dietary patterns, eating behavior, nutrition, diet

## Abstract

Functional dyspepsia is a gastrointestinal disorder characterized by postprandial fullness, early satiation, epigastric pain, and epigastric burning. The pathophysiology of the disease is not fully elucidated and there is no permanent cure, although some therapies (drugs or herbal remedies) try to reduce the symptoms. Diet plays a critical role in either the reduction or the exacerbation of functional dyspepsia symptoms; therefore dietary management is considered to be of high importance. Several foods have been suggested to be associated with worsening functional dyspepsia, such as fatty and spicy foods, soft drinks, and others, and other foods are thought to alleviate symptoms, such as apples, rice, bread, olive oil, yogurt, and others. Although an association between functional dyspepsia and irregular eating habits (abnormal meal frequency, skipping meals, late-night snacking, dining out, etc.) has been established, not many dietary patterns have been reported as potential factors that influence the severity of functional dyspepsia. A higher adherence to Western diets and a lower adherence to FODMAPs diets and healthy patterns, such as the Mediterranean diet, can contribute to the worsening of symptoms. More research is needed on the role of specific foods, dietary patterns, or specific eating habits in the management of functional dyspepsia.

## 1. Introduction

Functional dyspepsia (FD) is considered to be one of the most common disorders in clinical practice [1]. It has a high prevalence that affects 10–30% of adults and 3.5–27% of children worldwide [2]. Despite its high prevalence, there are major uncertainties regarding its definition, pathophysiology, diagnosis, treatment, and prognosis [1].

### 1.1. Pathophysiology

**Figure 1:**
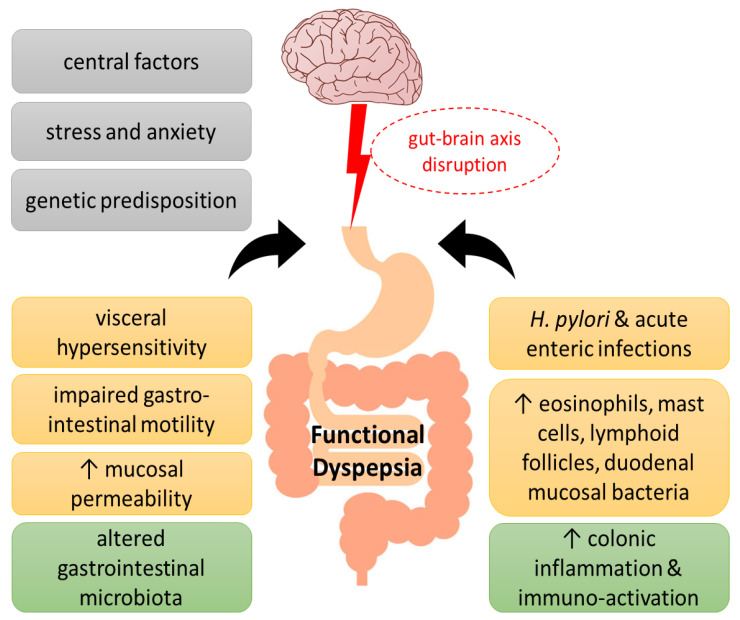
Pathogenesis of functional dyspepsia (FD). A series of pathogenic factors have been proposed for FD, including central nervous system abnormalities and genetic predisposition, as well as psychological factors, which have been suggested to interfere with the gut–brain axis function. Visceral hypersensitivity, impaired gastrointestinal motility, increased epithelial barrier permeability of the duodenal mucosa and infections, such as *Helicobacter pylori*, have been associated with altered intestinal flora in FD towards immune activation, immune cell infiltration, and low-grade inflammation.

Although the etiology of the disorder has not been fully elucidated, the main pathophysiological mechanisms that have been proposed throughout the years include motility alterations and psychosocial factors. The disruption of the microbiota–gut–brain axis, with abnormal central modulation, visceral hypersensitivity, and increased mucosal permeability contribute to the pathophysiology of FD. Increased intestinal permeability, immune activation, and gut dysbiosis caused by stress, which in turn affect the nervous system, suggests the concept of an impaired bidirectional communication of the “brain–gut axis” in FD [3]. In addition, acute enteric infections lead to colonic inflammation, recruitment of eosinophils and mast cells, lymphoid follicles, and duodenal mucosal bacterial loads, which affect the symptomatology of FD patients. Increased levels of inflammatory cytokines in the colonic mucosa are associated with anxiety and depression, which are related to the gut–brain axis [4]. Finally, genetic factors (such as polymorphisms in the genes related to gastrointestinal mobility and immune function), Helicobacter pylori infection, and impaired duodenal mucosal barrier function have been linked with worse FD symptoms [5,6,7] (Figure 1).

### 1.2. Clinical Manifestations

According to the Rome IV criteria for the diagnosis of functional dyspepsia, the main clinical symptoms include bothersome postprandial fullness, early satiation, epigastric pain, and/or epigastric burning along with the absence of any structural disease that may explain the symptoms. Furthermore, the above symptomatology impairs the patient’s quality of life and emotional health, and creates significant financial burden due to increased medical expenses and reduced work productivity. [8]. FD symptoms must be present for a minimum of 3 days a week during the last 3 months, they must be chronic, and start at least 6 months before diagnosis [9]. FD diagnosis includes an evaluation of the clinical history, physical examination, minimal laboratory tests, and a normal upper endoscopy. It is further categorized into epigastric pain syndrome (EPS) and eating-related postprandial distress syndrome (PDS). PDS is defined by bothersome postprandial fullness, that can affect typical activities, and/or bothersome early satiation, that can prevent the completion of a regular-sized meal. EPS is defined by bothersome epigastric pain and/or epigastric burning, both severe enough to disturb usual activities. The Rome IV classification involves not only PDS and EPS, but also their overlap (PDS-EPS overlapped syndrome), which is observed more frequently in hospital than in the general population [8].

### 1.3. Medicines

As there is no standard treatment for FD, research on effective therapies is ongoing, but still needs further confirmation. The acid-suppressive therapy with proton pump inhibitors (PPIs) is the most common treatment method [10]. Treatment with the tetracyclic antidepressant mirtazapine improves the quality of life, and buspirone, a serotonin-1A receptor agonist, can alleviate FD symptoms [11]. Prokinetics facilitate the gastric emptying rate [12], while amitriptyline, a neuromodulator, seems to be less effective for the treatment of FD [13]. Finally, the antibiotic rifaximin can change the duodenal microenvironment and reduce FD symptoms [14].

### 1.4. Herbal Remedies

Several herbal remedies have been proven effective and safe in FD with comparable outcomes with conventional treatments, and can serve as complementary and alternative medicine, especially when first line therapeutic approaches fail or are inaccessible to patients [9]. Some herbal oils improve PDS and EPS, and improve gastrointestinal symptom rating scale (GSRS) numbers and quality of life scores [15]. Herbal treatments show anti-inflammatory effects and contribute to an improvement in the function of gut microbiota, immune system, central stimuli, and intestinal motility in FD [16]. A systematic review and meta-analysis of 23 randomized controlled trials (RCTs) comparing the effectiveness of herbal treatments versus a placebo or other standard treatments for FD found that the majority of participants (>60%) in the herbal treatment group experienced an improvement in symptomatology and quality of life, compared to participants in the placebo group [17]. Chinese herbal medicines have been considered an effective alternative to prokinetics, according to a meta-analysis of 28 RCTs showing that Chinese herbal remedies were more effective than prokinetics at reducing the overall symptoms [18]. A combination of three herbs (*Trachyspermum ammi* L., *Anethum graveolens* L., and Zataria multiflora Boiss) may be important in the treatment of FD, as the essential oils were proven more effective than omeprazole [15]. Similarly, the Japanese Yukgunja-tang, also known as Rikkunshito, is a mixture of eight herbs that is frequently prescribed in FD [19], and it was proven more effective in the total clinical efficacy rate in a meta-analysis of 10 studies with 1246 patients, when combined with Western medicine over the use of Western medicine alone [20]. Additionally, perilla/ginger nutraceuticals have been shown to ameliorate some FD symptoms, such as epigastric pain, heartburn, and gastric reflux, with minor adverse events [21]. Artichoke leaf extract supplementation resulted in a greater amelioration of the multiple correspondence analysis scale compared to a placebo [22], while ginger accelerated gastric emptying [23]. The use of peppermint and caraway oil, a combination with unique properties, showed a statistically significant effect in the global improvement of FD symptoms in a meta-analysis of five RCTs [24]. A unique Greek herbal remedy known as Chios mastic gum has been shown to alleviate the symptoms of FD when taken daily for three weeks over a placebo [25]. The Hong Kong index of dyspepsia was used to assess the efficacy of the mastic treatment.

The current literature review was conducted with the aim of mapping the development of research on the nutritional aspects of FD during the period beginning on 1 January 2010 and ending on 31 December 2022. The research studies used fulfilled the following criteria: (1) they assessed the impact of foods, dietary patterns, eating behaviors, and botanicals on FD, including the relief of FD symptoms as the main outcome, (2) the literature was published in English, and (3) published from 2010 onwards. Duplicate/in vitro/animal studies, book chapters, study protocols, case reports, comments, and letters were excluded, as well as studies that involved multifactorial lifestyle interventions, in which the effects of dietary factors could not be distinguished from either genetic or lifestyle factors. To be accurate and trustworthy, the literature search was carried out using the PubMed-MEDLINE and Scopus databases. The search strategy included the following keywords: “Functional dyspepsia”, “Epigastric pain syndrome”, “Postprandial distress syndrome” “Nutrition”, “Diet”, “Foods”, “Dietary patterns”, “Dietary intervention”, “Nutrients”, “Macronutrients”, and “Micronutrients”. References from the extracted articles and reviews were also used to complete the data bank. The relevance of the studies was based on the title, abstract, and the full manuscript, which was reviewed. The search for duplicate publications was performed using an electronic database, and then the full text of each potentially relevant study was reviewed to ensure that it was consistent with the search criteria. A specific table was constructed by the same independent reviewers to facilitate the data extraction and selection, including the following information: article title, authors’ names, year of publication, participants’ characteristics, study design, duration of intervention, type of intervention, study outcomes, major findings, and limitations. Additionally, the studies were grouped according to their design (i.e., food-based intervention and dietary counseling interventions), and secondly according to the outcome.

## 2. Certain Foods as Inducers or Suppressants of Functional Dyspepsia Symptoms

Food consumption is the main triggering factor for dyspepsia in most FD patients. Several studies have reported that the consumption of specific foods may trigger or suppress FD symptoms. Most commonly reported triggering foods include fatty and spicy foods, soft drinks, wheat products, products containing caffeine, and alcohol [9]. Studies investigating foods that may trigger or alleviate symptoms of FD are presented in Table 1.

In the study of Akhondi-Meybodi and colleagues, three hundred and eighty four patients with FD, who had previously undergone endoscopy, were examined for their response to 114 foods in terms of relief or aggravation of their symptoms. Symptoms were most aggravated by sausage and bologna, pickles, vinegar, soft drinks, grains, tea, salt, pizza, watermelon, red pepper, and macaroni, as well as soft drinks, and acidic fruits [26]. Moreover, the foods that most frequently led to an alleviation of symptoms were apples, rice, rock candy, bread, caraway seeds, dates, honey, yogurt, quince, and walnuts. The evidence that particularly spicy foods stimulate FD symptoms has been confirmed by other studies. A spicy food’s burning sensation is caused by capsaicin found in high concentrations in chili peppers. Patients with FD who consume capsaicin-containing foods experience more symptoms than healthy controls or those who consume placebos [27,28]. It has been reported that transient receptor potential vanilloid-1 receptors (TRPV1) interact with capsaicin to cause the burning sensations, however the G315 polymorphism of the TRPV1 gene is inversely correlated with FD [29]. The prevalence of FD subtypes did not vary with the consumption of spicy foods or TRPV1 genotypes in a comparison of symptom generation according to these two factors, and TRPV1 polymorphisms were not associated with scores on symptom severity questionnaires, but eating spicy food was associated with higher scores for retching and stomach fullness [30]. When comparing the nutritional habits of 168 adults with FD to 135 healthy control subjects, spicy, but also fatty foods, and carbonated drinks were the most frequently reported food items to cause symptoms. In postprandial distress syndrome FD, symptoms were more likely to be brought on by carbonated beverages and legumes [31]. Similarly, the consumption of spicy, hot, raw, or cold foods, and dairy foods or products has been associated with FD symptoms, while tea was associated with FD prevention [32]. Canned foods, fast foods, and alcoholic beverages have also been linked with FD symptoms [33].

Patients with FD commonly report food hypersensitivities to wheat and gluten, a protein found in wheat. According to an Australian population-based study [34], 29% of people with FD avoided gluten, and self-reported gluten sensitivity had a significant association with FD diagnosis. In some cases, gluten-free diets have helped FD patients to improve their symptoms. Furthermore, in a randomized double-blind placebo-controlled trial in FD patients, the application of a gluten-free diet resulted in an improvement of symptoms in 35% of patients, yet only 18% of those patients were confirmed to have this sensitivity to gluten, with symptoms reoccurring following a blind gluten challenge [35]. This suggests that other components in wheat-based foods, such as fructans, may induce FD symptoms.

In sixty patients with FD, a 1-month retrospective food consumption frequency questionnaire was used to examine fifty-one foods that may stimulate FD symptoms [36]. The consumption of broccoli, radish, celery, green olives, and olive oil was lower in patients with increased postprandial fullness. Participants who had more pain in the stomach reported a lower consumption of dried fruits, green olives, butter, fast food, and alcohol, but the consumption of sunflower oil was higher. Overall, foods high in fat are often blamed by patients with FD for making their symptoms worse. Unknown pathophysiological mechanisms are responsible for high-fat foods’ ability to cause FD symptoms. However, cholecystokinin seems to be an important mediator [37]. Consuming fatty foods can slow down gastric emptying, disrupt gastric motility, and make dyspeptic patients feel fuller after meals [38]. Despite the known evidence regarding fatty foods, almonds do not appear to worsen FD symptoms as expected [38]. This effect may be related to the high content of tryptophan, a precursor of serotonin. Serotonin (5-hydroxytryptamine) is a key neurotransmitter involved in the regulation of gastrointestinal motility and sensory function. Indeed, stimulation of 5-HT1 serotonergic receptors induces smooth gastric muscle contractions that enhances gastric emptying, and appears to improve abdominal symptoms in patients [39]. However, when examining the brain activity using functional magnetic resonance imaging (fMRI) following the consumption of high- and low-fat foods with accurate or inaccurate fat information, researchers discovered that FD patients displayed more pronounced FD symptoms than healthy controls. These symptoms were less relieved following the consumption of high-fat yogurt than low-fat yogurt, regardless of the actual fat content. This suggests that low-fat foods may have a placebo effect or that high-fat foods may have a nocebo effect on symptom expression [40]. Interestingly, extra-virgin olive oil enriched with probiotics or antioxidants incorporated blindly into the regular diet of subjects with FD for seven days, induced a significant improvement in dyspeptic symptoms in those receiving the probiotic- or antioxidant-enriched oil diet, with the probiotic-enriched oil having a greater impact [41].

The relationship between coffee consumption and gastroesophageal reflux has been widely investigated [42,43,44]. Coffee has been considered a beverage that should be avoided, as it has been shown to worsen the symptoms of FD [8,26]. Chinese people with FD have been reported to be more likely to have a preference for coffee [45]. In the study by Correia et al., the effects of removing and substituting caffeinated or decaffeinated coffee with a non-caffeinated coffee alternative in the diet of fifty-one patients with FD were investigated [46]. Using a self-reported questionnaire, this descriptive, quasi-experimental pre/post intervention study looked at the relationship between functional dyspepsia and non-caffeinated coffee consumption. A statistically significant reduction in FD symptoms was reported following a month of consuming the coffee substitute.

## 3. Dietary Patterns and Eating Behaviors; Proportions, Variety, Frequency, and Cooking Habits

The dietary patterns and behavioral parameters examined constitute a multidimensional construct, including diet composition spanning from alimentary selection to the subsequent development of healthy or disordered eating habits, and ranging from meal frequency and regularity to cooking techniques. To the best of our knowledge, only a small percentage of studies provide a solution for to what extent do specific dietary behaviors influence and ultimately cause susceptibility to dyspeptic symptoms (Table 2).

Despite the difficulty of adhering to a FODMAP diet, Staudacher et al. noticed that individuals with underlying symptoms of FD and coexisting irritable bowel syndrome, and who followed a diet limited to FODMAPs, showed a higher reduction in the epigastric and total symptom score in comparison with those who received personalized dietary counseling at the discretion of the dietitians who participated in the research [47]. Similarly to Staudacher et al., Goyal and his team studied the effects of a low FODMAP diet in a panel of people with persistent FD, and subsequently they compared them to a slightly restricted standard diet that included an avoidance to spices, soft drinks, tea, coffee, and alcohol. Both interventions showed a significant remission of symptom severity and quality of life improvement, although one sub-group analysis of volunteers with postprandial distress syndrome or bloating were better responders to a low FODMAP regime [48].

Schnabel et al., within the NutriNet-Santé prospective observational cohort study, examined the interaction of consuming ultra-processed foods in people with various functional gastrointestinal disorders. Results showed that subjects suffering from FD and concurrent irritable bowel syndrome have a higher risk of being affected because of the increased daily amount of ultra-processed foods consumed [49]. In 2034 children and adolescents with functional gastrointestinal disorders, including FD, approximately 90% of the subjects reported fast food consumption, and there was a significantly higher prevalence of a history of functional disorder in fast food consumers, compared to non-consumers [50]. The increased risk of functional disorders was associated with the regular fast food intake. Ultra-processed and fast foods consist of the core of the Westernized dietary pattern. The popularity of ultra-processed and fast foods is partly due to their inexpensive and easy to find nature. However, the high fat content, particularly the trans fatty acid content and the presence of additives or reaction products owed to processing [51,52,53,54] may be linked with the augmentation of FD symptoms. Similarly, the prevalence of FD in Asia has increased during the last decades due to the change towards a more Westernized diet, higher in fat- and lower in carbohydrate-rich foods, whereas the spicy foods in Asian diets are associated with a higher risk of developing FD [55].

Quite the opposite, a dietary pattern that appears to be closely related to FD symptom amelioration is one that contains fruits and vegetables. According to the cross-sectional study by Tabibian et al., people on a diet rich in fruit have a 32% lower risk of FD, as well as a lower risk of early satiation and postprandial fullness in comparison with those on a low fruit diet. Moreover, a high vegetable intake seems to have a beneficial effect on FD, however, only in male subjects [56]. The same pattern was observed by Zito and his associates, who also emphasized through their results the importance of adopting a balanced diet, such as the Mediterranean diet due to its preventive function against FD. Specifically, a low adherence to the Mediterranean diet is associated with FD mainly in younger people [57].

Regarding the eating habits of FD subjects, a confusion about the frequency of main meals and snacks per day prevails in the bibliography. Göktaş et al. did not manage to demonstrate a significant difference in the frequency of main meals among FD and control subjects, as both groups had three main meals per day (68.5% and 70.4%, respectively). Likewise for snack consumption, researchers found no difference in their frequency between the two groups [31]. These findings corroborate those of Çolak et al., who recently extrapolated the conclusion that meal frequency had no impact on the triggering of FD symptoms among FD subjects [36]. Moreover, in a study involving 1139 volunteers, of whom 936 were healthy subjects and 203 had been diagnosed with FD, Xu et al. [45] demonstrated that the vast majority of the latter had by far more unhealthy nutritional habits than the control group (75.86% versus 37.50%, respectively; *p* < 0.001). It appears that the number of meals during the day shows an inversely independent relationship with the occurrence of FD. Hassanzadeh et al. [58] concluded that regularly consuming at least three main meals per day was not only indissolubly related with a 52% lower chance of developing FD compared to eating one meal, but also was inversely associated with the prevalence of early satiety. Similar findings were observed in Yamamoto et al.’s [59] cross-sectional study, where the risk of FD in subjects who ate one, two, and three meals daily was 4.8%, 2.2%, and 1.7%, respectively.

Correspondingly, the literature review shows that, three to five snacks per day are found to be a determinant factor for low FD incidence data, inhibiting alongside both the occurrence of postprandial discomfort (42%) and epigastric pain symptoms (43%) [58].

Concerning late-night snacking, the findings are controversial, with Xu et al. arguing that it is an independent triggering factor of postprandial fullness, while Yamamoto et al. state that there is no correlation with the development of FD. Carvalho et al. claim that the duration of the overnight fast was significantly longer in subjects with FD than in the controls, as the former tended to eat dinner earlier. On the contrary, this difference is equated to daytime fasting [38].

Based on the current literature, another causative agent for the generation of dyspeptic symptoms is the skipping of one or even more main meals during the day, and depending on which meal (breakfast, lunch, dinner), there are different impacts on FD and its subtypes. In particular, Yamamoto et al. claim that FD subjects were more prone to skip lunch and dinner than the controls, and the habit of omitting breakfast and/or lunch was independently inversely related to a high incidence of FD symptoms (breakfast: adjusted OR, 1.60 [95% CI, 1.10–2.32] and lunch: adjusted OR, 2.52 [95% CI, 1.04–5.18]). Contrarily, researchers did not prove any relation between FD prevalence and skipping dinner [59]. A more comprehensive description can be found in Xu et al.’s study, that not only confirms the analogous findings, but also reveals the close association between skipping breakfast and PDS (18/203) and EPS (13/203) subtypes, but not with their overlapping type (9/203) [45].

Apropos irregular meals and the tendency of dining out, which are positively connected to the generation of FD symptoms, investigators claim that both correlated with PDS, but only an erratic dinner time was related to EPS and the overlapping subtype [45]. Furthermore, in respect to the possible correlation between sleep and the development of FD symptoms, researchers did not prove any statistically significant difference between FD and control subjects who go to bed following a meal [38].

**Figure 2:**
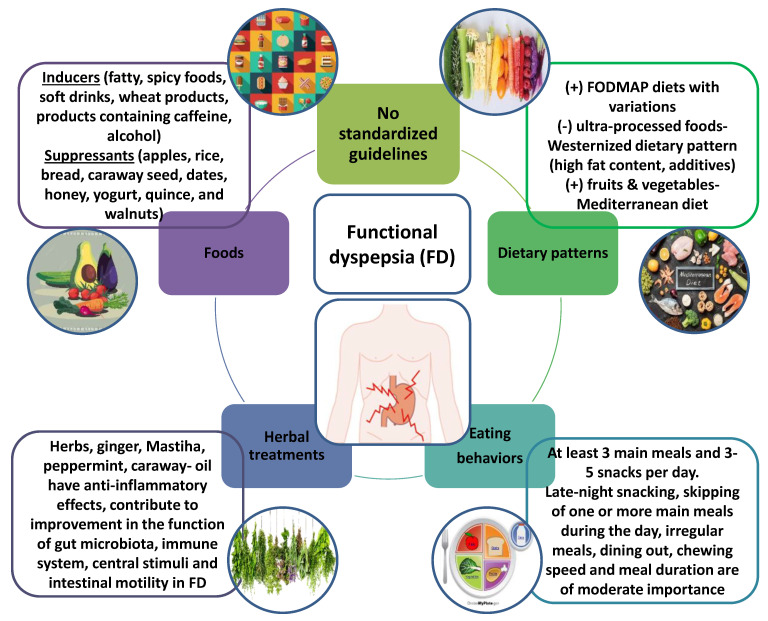
Functional dyspepsia (FD) is considered one of the most common disorders in clinical practice. Standardized guidelines for the nutritional approach and eating habits of patients with FD do not exist. However, assessing the impact of foods, dietary patterns, eating behaviors and botanicals on FD can help, on the one hand, to elucidate the pathophysiology of the disease, and on the other hand, to improve palliative treatments.

In regard to meal time or chewing efficiency, and their role in triggering FD symptoms, Çolak et al. [36] claim that this assertion has no merit, although some clinical studies demonstrate that subjects with FD are more likely to self-report themselves as fast eaters than the controls [38,60]. In the same cross-sectional study, the findings indicated that roasting was the most usual cooking method in subjects experiencing the postprandial fullness symptoms [36]. Furthermore, compared to both PDS and EPS subtypes, as well as the control group, the overlapping group appeared to have a significantly higher intra-meal fluid consumption [31] (Figure 2).

## 4. Conclusions

Well-structured and standardized guidelines for the nutritional approach and eating habits of patients with FD do not exist, and randomized controlled trials are few, while most available evidence comes from observational studies. Hence, although the contribution of specific foods identified as triggers by FD patients varies, some causal relationships between specific foods and symptoms have been demonstrated. The retrospective nature of most studies and the lack of a standardized method for verifying the food-symptom association accounts for the difficulty in accumulating a definitive list of foods to avoid or foods to favor. Some of the most frequently reported foods to avoid are fatty foods, processed foods, and wheat products. Alike, processed foods and overall the Western dietary pattern, which includes fatty foods, as these are considered inducers of FD symptoms. Eating small and frequent meals may be a reasonable suggestion to reduce symptoms, however the evidence is inadequate and inconsistent. In the future, the improvement in our understanding of the pathophysiological mechanisms underlying FD might lead to well-designed and target-driven clinical trials on the effects of foods, dietary patterns, or specific eating habits in the management of FD.

## Figures and Tables

**Table 1:** Studies investigating foods that may trigger or alleviate symptoms of FD.

a/a	Ref. No	Foods/Nutrients Investigated	Design	Results	FD Symptoms
1	[26]	Commonly consumed foods	Observational study; Three-hundred and eighty-four patients with an FD diagnosis (Rome III diagnostic criteria), aged 39.16 ± 14 years, 39.6% men.	Sausage and bologna, pickled foods and fruits, vinegar, soft drinks, grains, tea, salty foods, pizza, watermelon, red pepper, macaroni (pasta), and fatty oils;	↑
				Apples, rice, rock candy, bread, caraway seeds, dates, honey, yogurt, quinces, almonds, and walnuts.	↓
2	[27]	Capsaicin containing capsule	Randomized, double-blind, placebo-controlled trial; Seventy-three patients with FD (Rome II diagnostic criteria), aged 39.3 ± 11.0 years, 26% men; Intervention group (*n* = 42) received a capsaicin containing capsule (0.75 mg), and placebo group (*n* = 31).	Capsaicin vs. placebo group:	
				✓ Positive test in upper gastrointestinal symptoms;	↑
				✓ Median symptom score.	
3	[28]	Capsaicin containing capsule	Case-control study: 61 healthy controls and 54 FD patients (Rome II diagnostic criteria) received a capsaicin containing capsule (0.75 mg); Placebo case-control study: 19 healthy controls and 13 FD patients (Rome II diagnostic criteria) received a placebo capsule.	FD patients vs. controls and vs. placebo:	
				✓ Positive test in upper gastrointestinal symptoms;	↑
				✓ Median symptom score.	
4	[29]	Capsaicin	Case-control study 98 subjects with no upper abdominal symptoms and 109 patients with FD (Rome III diagnostic criteria)	Transient receptor potential vanilloid-1 receptors (TRPV1) in FD;	↑
				The G315 polymorphism of the TRPV1 gene is inversely correlated with FD.	
5	[30]	Spicy foods	Observational study; One-hundred ant twenty-one FD patients (Rome III diagnostic criteria) of whom 35 carried TRPV1 CC and 28 carried GG genotypes.	Stomach fullness and retching regardless of genotype.	↑
6	[31]	Commonly consumed foods	Case-control study; A total of 168 adults with FD (Rome III diagnostic criteria) and 135 healthy controls; FD patients were categorized into epigastric pain syndrome (EP-FD), postprandial distress syndrome (PS-FD), and mixed (MX-FD) subgroups.	FD patients vs. controls:	↑
				✓ fried and fatty foods, hot spices, and carbonated drinks.	
				Carbonated drinks in PS-FD group vs. other subgroups.	↑
7	[32]	Commonly consumed foods	Case-control study; Seven-hundred and fifty-nine university students categorized into the FD (Rome III diagnostic criteria) group (*n* = 128) and healthy group.	FD patients vs. controls:	
				✓ Spicy, hot, raw, or cold foods and dairy.	↑
				Tea was associated with FD prevention.	
8	[33]	Commonly consumed foods	Observational study; One-hundred and eighty-four subjects participated in a 4-month study; FD (Rome III diagnostic criteria) was present in 7.6%, and gastroesophageal reflux disease was present in 31.0%.	Canned foods, fast foods, and alcoholic beverages in FD.	↑
9	[34]	Wheat	Observational study; A total of 3542 people were randomly selected from the Australian population.	Self-reported wheat sensitivity in FD (Rome III diagnostic criteria).	↑
10	[35]	Gluten	Observational study: 77 patients with refractory FD followed a gluten-free diet for 6 weeks; Patients with ≥30% improvement (*n* = 27) participated in a randomized double-blind placebo-controlled crossover trial: the intervention group (*n* = 14) received gluten-free muffins for one week and the control group received gluten muffins for one week.	Gluten free diet (observational study):	
				✓ Gastrointestinal symptoms;	
				- Sixty-five percent did not respond, while 35% cases showed gastrointestinal symptoms improvement.	↔
				Gluten challenge (randomized trial):	
				- Symptoms recurred in five cases suggesting the presence of non-celiac gluten sensitivity.	↔
11	[36]	List of foods that may stimulate FD	Observational study; Sixty patients with FD followed a gluten-free diet for 6 weeks (Rome IV diagnostic criteria).	Consumption of broccoli, radish, celery, green olives, and olive oil in subjects with postprandial fullness;	↓
				Consumption of alcohol, dried fruits, green olives, butter, and fast food in subjects with stomach pain;	↓
				Consumption of sunflower oil in subjects with stomach pain .	↓
12	[38]	Commonly consumed foods	Case-control study; Forty-one patients with FD (30 women, 11 men; mean age: 46 ± 12 years) and 30 healthy volunteers (25 women, five men; mean age: 35 ± 12 years).	FD patients vs. controls:	
				✓ Carbonated drinks, fried foods, red meat, sausage, coffee, pasta (macaroni, lasagna), milk, cheese, sweets, pepper, bananas, pineapple, cucumber, orange, beans, bread, and spicy foods;	↑
				✓ Heartburn: pepper and coffee;	↑
				✓ Bloating: carbonated drinks, onions, beans, and bananas;	↑
				✓ Epigastric burning: coffee, cheese, onions, pepper, milk, chocolate, and pineapple;	↑
				✓ Fullness: red meat, bananas, bread, cakes, pasta, sausage, fried foods, beans, mayonnaise, milk, chocolate, eggs, sweets, and oranges.	↑
13	[40]	Four different yogurts differentiated in fat composition label	Case-control cross-over study; Twelve FD (Rome III diagnostic criteria) patients (five men, aged 46.46 ± 5.64 years) and 14 age- and body mass index-controlled healthy subjects (five men, aged 45.79 ± 4.71 years); Subjects consumed four different yogurts during four separate visits: high-fat yogurt with “high fat” label (HH), high-fat yogurt with “low fat” label (HL), low-fat yogurt with “high fat” label (LH), and low-fat yogurt with “low fat” label (LL).	FD patients vs. controls:	
				✓ Burning, discomfort, pain, bloating, nausea, and fullness at baseline;	↑
				✓ Satiation, discomfort, burning, and abdominal pain for high fat label vs. low fat label;	↑
				✓ Amplitude of low-frequency fluctuations (ALFFs) regardless of the type of yogurt consumed;	↑
				✓ Functional connectivity from the insula to the occipital cortex (I-O) after high fat ingestion;	↑
				✓ Functional connectivity from the insula to the occipital cortex (I-O) after low fat ingestion;	↓
				✓ Functional connectivity from the insula to the precuneus (I-P) after ingestion of low fat–labeled yogurt.	↑
				In FD patients:	
				✓ I-O functional connectivity was negatively correlated with nausea;	
				✓ I-P functional connectivity was negatively correlated with FD symptom intensity, food craving, and depression.	
14	[41]	Extra virgin olive oil enriched with antioxidants or probiotics	Randomized controlled trial; Eight FD patients (Rome III diagnostic criteria); Each subject received two vials of 9 mL (equal to the daily food requirement) per day either of: (a) extra virgin olive oil, (b) extra virgin olive oil enriched with antioxidants, or (c) oil enriched with probiotics, to be added to the meals for 7 days.	Probiotic olive oil vs. plain olive oil:	
				✓ Nausea;	↓
				✓ Pain/discomfort.	↓
				Probiotic vs. antioxidant rich olive oil:	
				✓ Belching;	↓
				✓ Postprandial gastric distension and fullness.	↓
				Probiotic olive oil was more effective than antioxidant olive oil.	
15	[45]	Commonly consumed foods	Observational study; A total of 1304 adults residents were recruited: 165 had existing organic dyspepsia, 203 were diagnosed with FD (Rome III diagnostic criteria), and the other 936 were healthy controls.	FD patients vs. controls:	
				✓ Consumption of fatty foods, sweets, and coffee.	↑
16	[46]	Non-caffeinated coffee substitute	A quantitative study; Fifty-one patients, aged 29–83 years, and diagnosed with FD; Each participant received a commercially available 7- ounce bottle of a non-caffeinated coffee substitute, consisting of roasted barley as the main ingredient with roasted malt barley, roasted chicory, and roasted rye; Participants were instructed to substitute their usual daily coffee consumption with the non-caffeinated coffee substitute for 1 month.	Post vs. pre-intervention:	
				✓ Dyspepsia symptoms;	↓
				✓ Reflux, indigestion, diarrhea, and constipation.	↓

**Table 2:** Studies investigating dietary patterns and eating behaviors associated with FD.

a/a	Ref. No	Dietary Pattern/Eating Behavior	Design	Results	FD Symptoms
1	[47]	Low FODMAP	Randomized-controlled study; Fifty-nine patients with FD (Rome IV diagnostic criteria); Individuals received either low FODMAP (*n* = 40) or standard dietary advice as per clinical judgment from the consulting dietitian (*n* = 19).	Low FODMAP vs. control:	
				✓ Epigastric score;	↓
				✓ Total symptom score.	↓
2	[49]	Ultra-processed food rich diet	Observational study; A total of 44,551 adults (> 45 years) from the French NutriNet-Santé Study.	Dietary factors associated with FD:	
				✓ Ultra-processed food rich diet.	↑
3	[50]	Fast food diets	Observational study; A total of 2034 adolescents (aged 12–19 years) from the Nutrition and Health Survey in Taiwan (NAHSIT) with functional gastrointestinal disorders (Rome III diagnostic criteria).	Dietary factors associated with FD development:	
				✓ Fast foods;	↑
				✓ Low fiber intake and frozen desserts in the diet.	↑
4	[56]	Fruit and vegetable intake	Observational study; A total of 3362 middle-age participants of whom 14.5% was diagnosed with FD (Rome III diagnostic criteria).	Dietary factors associated with FD development:	
				✓ Fruit consumption;	↓
				✓ Vegetable consumption.	↔
				Fruit consumption was associated with a lower risk of early satiation.	
5	[57]	Mediterranean diet	Observational study; A toatl of 1134 subjects (age 17–83 years); Seven-hundred and nineteen (63.4%) were healthy controls, 172 (13.3%) patients had irritable bowel syndrome (IBS), and 243 (23.3%) had FD (Rome III diagnostic criteria).	Dietary factors associated with FD development:	
				✓ Low adherence to a Mediterranean diet.	↑
6	[31]	Eating behavior	Case-control study; A total of 168 adults with FD (Rome III diagnostic criteria) and 135 healthy controls; FD patients were categorized into epigastric pain syndrome (EP-FD), postprandial distress syndrome (PS-FD), and mixed (MX-FD) subgroups.	Symptomatology in FD patients vs. controls:	
				✓ Meal frequency;	↔
				✓ Snacking;	↔
				✓ Intra-meal fluid consumption.	↑
7	[36]	Eating behavior	Observational study; Sixty patients with FD followed a gluten-free diet for 6 weeks (Rome IV diagnostic criteria).	Symptomatology in FD patients vs. controls:	
				✓ Meal frequency;	↔
				✓ Roasting.	↑
8	[45]	Eating behavior	Observational study: A total of 1304 adults residents were recruited: 165 had existing organic dyspepsia, 203 were diagnosed with FD (Rome III diagnostic criteria), and the other 936 were healthy controls.	Dietary habits in FD vs. controls:	
				✓ Irregular mealtime, meal frequency, night snacking, skipping breakfast, and dining out.	↑
9	[58]	Meal frequency	Observational study; A total of 4763 individuals from the general adult population.	Dietary factors associated with FD development:	
				✓ >3 meals/day;	↓
				✓ Three to five snacks/day.	↓
10	[59]	Eating behavior	Observational study: A total of 8923 Japanese university students: 168 subjects had FD (Rome III diagnostic criteria) and 8745 were healthy controls.	Dietary factors associated with FD development:	
				✓ Skipping breakfast/lunch;	↑
				✓ Skipping dinner, extra meals (snacks), or midnight snacks;	↔
				✓ Frequency of meals.	↓
11	[38]	Eating behavior	Case-control study; Forty-one patients with FD (30 women, 11 men; mean age: 46 ± 12 years) and 30 healthy volunteers (25 women, 5 men; mean age: 35 ± 12 years).	Dietary factors associated with FD development:	
				✓ Overnight fast;	↑
				✓ Frequency of meals;	↓
				✓ Daytime fast, fast eating, or going to bed soon after the meal.	↔
12	[60]	Eating behavior	Observational study; Eighty-nine adult women: 11 subjects had FD (Rome III diagnostic criteria), six subjects with gastroesophageal reflux disease, and 72 healthy controls.	Dietary factors associated with FD development:	
				✓ Fast eating.	↑

## Data Availability

No new data were created.
